# Early-stage Hodgkin lymphoma in Cape Town, South Africa: prognostic risk factors at diagnosis and treatment outcomes

**DOI:** 10.1186/s12885-025-15219-0

**Published:** 2025-11-25

**Authors:** Shakira Dawood, Brigid McMillan, Karryn Brown, Jenna Oosthuizen, David Richardson, Kudakwashe Simba, Lillian F. Andera, Jessica Opie, Katherine Antel, Stuart More, Ayesha Allie, Zainab Mohamed, Estelle Verburgh

**Affiliations:** 1 Department of Medicine, University of Cape Town and Groote Schuur Hospital, Cape Town, South Africa; 2 Department of Radiation Oncology, University of Cape Town and Groote Schuur Hospital, Cape Town, South Africa; 3 Department of Medicine, Division of Clinical Haematology, University of Cape Town and Groote Schuur Hospital, Cape Town, South Africa; 4 Department of Pathology, Division of Haematology, National Health Laboratory Service, University of Cape Town and Groote Schuur Hospital, Cape Town, South Africa; 5 Division of Haematology and Oncology, Medical University of South Carolina, Charleston, USA; 6 Department of Radiation Medicine, Division of Nuclear Medicine, University of Cape Town and Groote Schuur Hospital, Cape Town, South Africa

**Keywords:** Lymphoma, Hodgkin disease, South Africa, Treatment outcome, Early diagnosis, NCCN staging

## Abstract

**Background:**

Classic Hodgkin Lymphoma (cHL) is a highly curable cancer disproportionately affecting younger patients and those with human immunodeficiency virus (HIV) infection. In under-resourced regions with endemic HIV or tuberculosis, cHL treatment outcomes are poor when compared to high-income settings for numerous disease, patient and health systems related factors. Over 80% of patients present with advanced disease and reducing diagnosis delays is thought to be critical in improving survival. However, because early-stage cHL is relatively uncommon in these settings, data evaluating outcomes in patients with early-stage disease are lacking.

**Method:**

This retrospective study examined 70 patients with early-stage cHL. These patients, derived from a consecutive cohort of 387 cHL patients treated between 2010 and 2022, were meticulously staged with the Modified Lugano staging system and positron emission tomography / computed tomography (PET/CT). Progression-free survival (PFS), overall survival (OS), and the applicability of National Comprehensive Cancer Network (NCCN) risk stratification were assessed.

**Results:**

The median age at diagnosis was 35 years and most patients had stage II (86%), or unfavourable disease (76%). The 5-year OS for the favourable and unfavourable groups were similar (94% vs. 90%, *P* = 0.599) as was 5-year PFS (83% vs. 88%, *P* = 0.984). Five-year OS for the HIV positive patients was 94% and 90% in HIV negative patients (*P* = 0.684), and PFS was 100% and 82%, respectively (*P* = 0.086).

**Conclusions:**

This study demonstrated survival outcomes in early-stage cHL patients comparable with higher income countries, notwithstanding HIV status or unfavourable NCCN classification. In contrast to the previous study from our centre which showed overall survival rates of 66%, for patients with cHL of all disease stages, we highlight the importance of facilitating early diagnosis and treatment in low- and middle-income countries.

**Supplementary Information:**

The online version contains supplementary material available at 10.1186/s12885-025-15219-0.

## Background

Classic Hodgkin lymphoma (cHL) is a unique neoplasm of the B-cell lymphocyte lineage characteristically affecting young people with a global incidence of 1–3 per 100,000 individuals per year [1]. In PLWH (people living with HIV), the risk of developing cHL is increased at least 7-fold even with antiretroviral therapy (ART) [2–6]. Patients with early-stage cHL (modified Lugano stage I and II) have an especially favourable cure rate exceeding 90% with first-line combination chemotherapy and radiotherapy, a cure rate continually improving over the past 75 years due to adjustments in chemotherapy and radiation therapy [7–13].

Patients with cHL in South Africa (SA) are at a distinct disadvantage because only a small proportion, 13% − 18%, of cHL patients are diagnosed with early-stage disease. In contrast, 52–59% of patients are in early-stage cHL at diagnosis in the United States [5, 14–17]. In HIV-endemic environments, the overwhelming numbers and overlapping symptomatology of tuberculosis is a major obstacle to recognising lymphoma. Inappropriate investigations and presumptive tuberculosis therapy prolong the diagnostic interval. Consequently, patients have advanced disease at diagnosis and poor survival [15, 18–20]. In two cohorts from our institution, inclusive of all cHL stages, we reported 5-year define OS of 56% and 66% - notably worse outcomes than those seen in high-income countries [15, 19].

A second consequence to these diagnostic delays, is limited data on early-stage cHL patient outcomes in SA. Likewise, prognostic stratification of early-stage cHL into favourable and unfavourable risk, has not been properly evaluated in our developing world context [7, 21, 22]. This due to both low patient numbers and limited access to the required testing modalities, most notably (PET/CT) [11, 17, 23, 24].

The aim of this retrospective study was to focus on the early-stage patient subset of the consecutive cHL cohort diagnosed and treated in our centre since 2010. We sought to determine progression-free survival (PFS) and OS in this subset and how this correlated with the NCCN staging system and its individual risk factors. We hypothesised that if early-stage cHL patient outcomes in our setting prove to be internationally comparable, it confirms that our primary priority for obtaining cure in cHL must be to address healthcare system delays.

## Materials and methods

### Study design and participant selection

Groote Schuur Hospital (GSH) is a 975-bed tertiary academic treatment centre in the Western Cape, South Africa, affiliated with the University of Cape Town, where patients without private medical insurance access state-funded healthcare. In the Western Cape region, patients 13 years and older are treated in adult units. All cHL patients diagnosed and/or treated in the GSH Radiation Oncology and Clinical Haematology units are entered into the Haematology Patient Registry (HPR) housed in the REDCap electronic database hosted by the university (HREC R024/2018). Patients from the HPR with stage I and II cHL consecutively diagnosed between 1 January 2010 and 31 December 2022 were selected for this study.

### Demographic and clinical data

Patient data were captured in the HPR drawing from hospital folders, the National Health Laboratory Service (NHLS), and the Western Cape Hospitals’ repositories for imaging data (IntelliSpaceVR, PACS Enterprise and NUcMEDVR systems) [25, 26]. These data included investigations for tuberculosis and HIV done in the period from symptomatic presentation to diagnosis with cHL (Supplementary Information: Methods and Materials).

Patients were treated based on NCCN and European Society for Medical Oncology (ESMO) guidelines, adapted according to local resource constraints. Patients received two cycles of ABVD (Adriamycin (doxorubicin), bleomycin, vinblastine, dacarbazine), followed by PET/CT and risk-adapted treatment. During the study period 2010 to 2022 NCCN guidelines were regularly updated and ESMO guidelines were published in 2014 and 2018 [7, 27]. Our local guidelines evolved based on international standards of care, and some adaptation was necessitated due to local resource constraints. Due to higher cost as well as added complexity of administration and increased risk of infection, escalated BEACOPP (bleomycin, etoposide, Adriamycin, cyclophosphamide, Oncovin (vincristine), procarbazine, prednisone) was not included in our protocol for patients with ESMO limited and intermediate stage cHL (NCCN stage I and II favourable and unfavourable) who had PET-positive disease after two cycles of ABVD. The definition of PET-positive in our protocol was Deauville score (DS) ≥ 3 as used in HD10 [28, 29]. International Lymphoma Radiation Oncology Group (ILROG), ESMO and NCCN guidelines were used to inform indications for radiotherapy [7, 27, 30, 31]. Patients with early-stage disease were managed with combined modality or a chemotherapy-only approach (Supplementary Table 1). Patients with very good risk disease according to the GHSG received two cycles of ABVD and 20 Gy involved site radiation therapy (ISRT). The patients with ESMO intermediate stage (or NCCN poor risk early-stage) disease received four cycles of ABVD and 30 Gy ISRT [7, 27].

Response assessment was based on the International Working Group criteria and incorporated regular updates from the NCCN guidelines into our clinical treatment regimens [7, 32]. Early interim PET/CT after two cycles of ABVD was used to assess response and guide further clinical management. Patients with a DS 1–3 completed three cycles of ABVD if they were assessed as having favourable disease or four cycles of ABVD if they had unfavourable disease as per ESMO Clinical Practice Guidelines [27]. Both groups received 30 Gy ISRT. Patients were treated with an ABVD-only regimen if they chose not to have radiotherapy, if radiotherapy was contraindicated or there were too many sites of disease to safely deliver radiotherapy, and if pre-chemotherapy imaging was inadequate. Prior to 2012, ISRT was delivered using 3D conformal radiotherapy, and from 2012 all patients are treated with Volumetric Arc Therapy. Patients with DS 4 with focal positivity were treated with 36 Gy ISRT after completing four cycles of ABVD, followed by an end of treatment PET/CT. Those with DS 4–5 at interim PET/CT with suspicion for refractory disease were discussed at a multidisciplinary team meeting involving radiation oncology and clinical haematology, referred for a biopsy if indicated, and offered high dose salvage chemotherapy and autologous stem cell transplantation as per the unit’s standard of care protocol.

### Statistical analyses

Data were analysed using STATA version 18.0 (Stata Corporation, College Station, Texas, USA). Categorical variables were described by frequencies (%) and numerical variables were described by medians (IQR: interquartile range) as data were non-parametric. OS and PFS were estimated by Kaplan-Meier curves and differences between NCCN risk groups and HIV status groups were tested by log-rank tests. OS was defined as the time from diagnosis to death from any cause, or last encounter (censored) at a public health facility in the Western Cape. PFS was defined as the time from diagnosis to disease progression, relapse, or last encounter. Univariable and multivariate logistic regression models were used to assess associations between covariates and HL failure (disease progression, refractory disease, or relapse). For all analyses a *p* value < 0.05 was considered statistically significant.

## Results

### Study population

There were 387 cHL patients in the HPR diagnosed between 1 January 2010 and 31 December 2022, of whom 70 patients (18% of total cHL) had stage I and II cHL and were selected for retrospective analysis. The cohort included early-stage cHL patients from previous publications from our institution: 13 diagnosed between January 2010 and December 2012 (15%; *n* = 85) published by Swart et al., and eight diagnosed between June 2012 and June 2014 (20%; *n* = 41) published by Antel et al. [15, 19]. In this study population, there was an equal male: female ratio. The median age of 35.4 years (IQR: 26.3–44.7; range: 15–63) was comparable between the HIV positive (51 patients, 73%) and negative cohorts (19 patients, 27%). No second peak in the elderly was seen (Table 1).

**Table 1:** Baseline demographic and clinical characteristics of the 70 early-stage cHL patients

	*N* (%) or Median (IQR)		
Variable	Total (*N* = 70)	HIV Negative (*N* = 51, 73%)	HIV Positive (*N* = 19, 27%)
Sex			
Male	35 (50)	27 (52)	8 (42)
Female	35 (50)	24 (47)	11 (58)
Age (years)	35 (26–45)	34 (26–45)	37 (28–48)
Clinical stage*			
I	10 (14)	5 (10)	5 (26)
II	60 (86)	46 (90)	14 (74)
Histological classification			
NSHL	41 (59)	34 (67)	7 (37)
MCHL	15 (21)	8 (16)	7 (37)
LRHL	1 (1)	1 (2)	0
LDHL	2 (3)	0	2 (11)
HL unspecified	11 (16)	8 (16)	3 (16)
Laboratory parameters			
Haemoglobin (g/dL)	12 (10–14)	12 (12–14)	11 (10–14)
Albumin (g/dL) (*n* = 50)	40 (38–44)	41 (38–44)	40 (37–43)
Lymphocyte count (x10^9^/L) (*n* = 67)	2 (1–3)	2 (2–3)	2 (1–2)
White cell count (x10^9^/L)	8 (6–11)	9 (7–13)	6 (5–8)
NCCN risk factors			
MMR ratio > 0.33			
Yes	7 (10)	7 (14)	0
No	63 (90)	44 (86)	19 (100)
Bulky disease			
Yes	10 (14)	8 (16)	2 (11)
No	60 (86)	43 (84)	17 (90)
ESR ≥ 50 mm/hr			
Yes	19 (27)	12 (24)	7 (37)
No	23 (33)	17 (33)	6 (31)
Unknown	28 (40)	22 (43)	6 (31)
B-symptoms present			
Yes	27 (39)	22 (43)	5 (26)
No	43 (61)	29 (57)	14 (74)
> 3 nodal regions involved			
Yes	28 (40)	24 (47)	4 (21)
No	42 (60)	27 (53)	15 (79)
NCCN classification			
Favourable	17 (24)	10 (20)	7 (37)
Unfavourable	53 (76)	41 (80)	12 (63)

*HL* Hodgkin lymphoma, *NSHL* nodular sclerosing HL, *MCHL* mixed cellularity HL, *LRHL* lymphocyte rich HL, *LDHL* lymphocyte depleted HL, *NCCN* National Comprehensive Cancer Network, *IQR* Interquartile range, *MMR* mediastinal mass ratio, *ESR* erythrocyte sedimentation rate

Investigations for tuberculosis were performed in 48 patients (69%), 32 (63%) people without HIV infection and 16 (84%) PLWH. Tuberculosis was confirmed in five, all of whom were (7% PLWH of the total cohort and 26% of PLWH). At the time of cHL diagnosis, three patients (4%) were on treatment for laboratory proven disease, and six patients (9%) were treated presumptively, one of these patients was subsequently proved to have multidrug-resistant tuberculosis. None of the nine patients receiving anti-tuberculosis therapy developed refractory or relapsed cHL.

### Prognostication

The NCCN scoring system variables are presented in Table 1. The commonest unfavourable characteristic was an elevated ESR (> 50 mm/hr) seen in half of those tested (45%, *n* = 19/42). The presence of B-symptoms or involvement of > 3 nodal regions were also frequently seen (39% and 40%, respectively). An increased MMR was not seen in any PLWH.

**Fig. 1:**
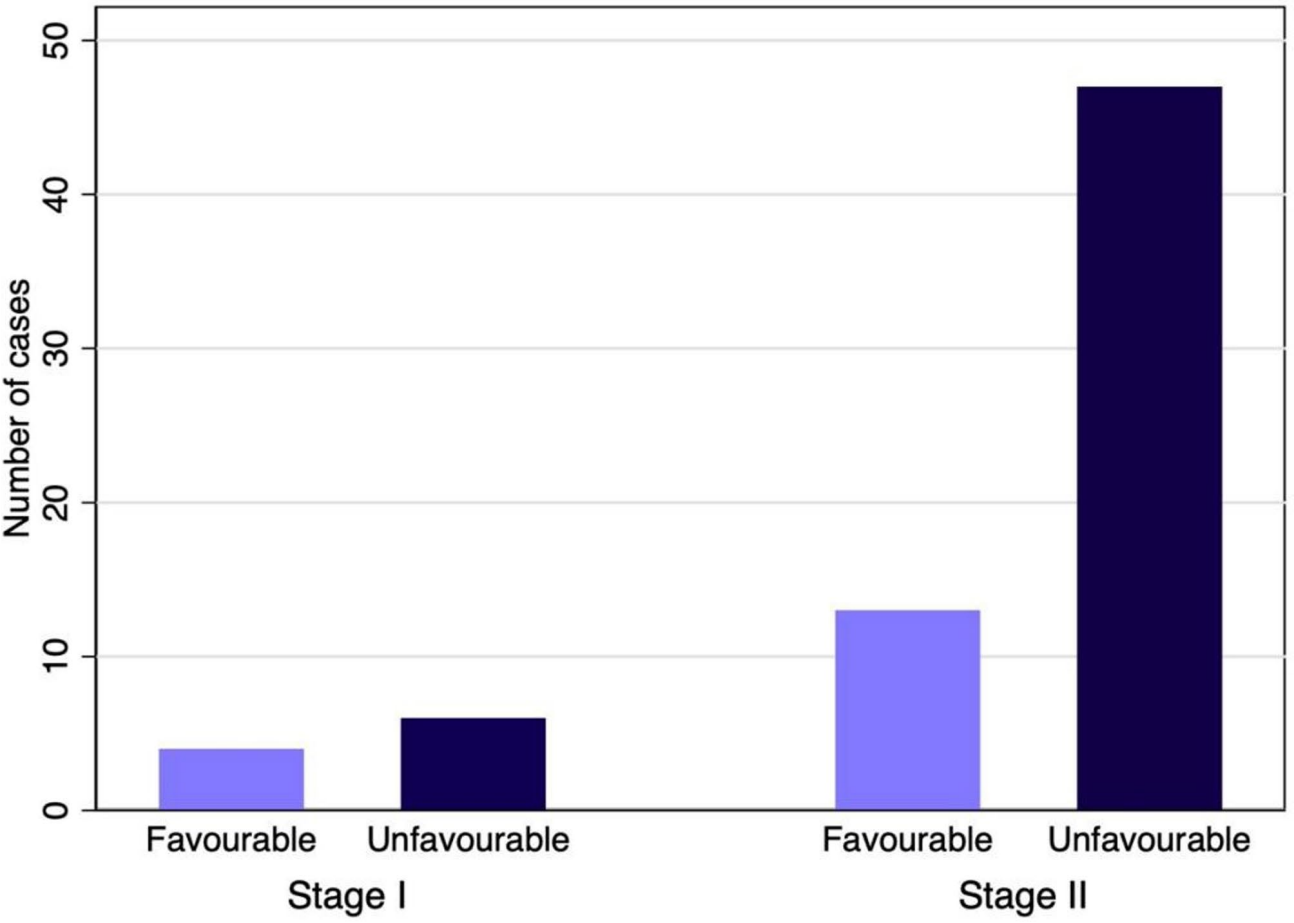
Distribution of patients according to clinical stage and NCCN classification (favourable and unfavourable)

At the time of diagnosis, ten patients (14%) had stage I disease, and 60 (86%) had stage II disease. Staging was based on PET/CT in 37 patients (53%) and on CT in the remaining 33 patients (47%). Most patients (76%) were classified as NCCN unfavourable early-stage HL. This included six (60%) of stage I, and 47 (78%) of stage II patients (Fig. 1). Compared with HIV negative patients, fewer PLWH had stage II disease, an MMR > 0.33, more than three nodal regions involved, and unfavourable disease, while more had an ESR ≥ 50 mm/hr.

### Treatment

Chemotherapy and ISRT was given as primary treatment to 36 patients (51%), while 34 patients (49%) received chemotherapy alone. The most common chemotherapy regimen used was ABVD, used in 93% of patients (Table 2).

**Table 2:** Treatment methods, patient responses and outcomes

	*N* (%)		
Variable	Total (*N* = 70)	HIV- (*N* = 51, 73%)	HIV+ (*N* = 19, 27%)
Primary treatment			
Chemotherapy	34 (49)	22 (43)	10 (53)
Combined modality chemotherapy & radiotherapy	36 (51)	29 (57)	9 (48)
First-line chemotherapy regimen			
ABVD	65 (93)	48 (94)	17 (90)
Other*	5 (7)	3 (6)	2 (11)
Response to primary treatment			
Complete response	60 (86)	45 (88)	15 (79)
Partial response	2 (3)	1 (2)	1 (5)
Progressive disease	4 (6)	4 (8)	0
Unconfirmed complete response	4 (6)	1 (2)	3 (16)
Relapse after primary treatment	3 (4)	3 (6)	0
Outcome			
Alive	64 (91)	46 (90)	18 (95)
Died	6 (9)	5 (10)	1 (5)
Cause of death			
Progressive disease	2 (3)	2 (4)	0
Early treatment interruption and refusal	1 (1)	1 (2)	0
Covid pneumonia 3 months post complete response	2 (3)	1 (2)	1 (5)
Fatal car accident 8 years post complete response	1 (1)	1 (2)	0

*Other chemotherapy includes: 1 EBVD (epirubicin, bleomycin, vinblastine, dacarbazine), 1 AVD (Adriamycin, vinblastine, dacarbazine), 1 ChIVPP (chlorambucil, vinblastine, procarbazine, prednisolone), 1 ChIVPP and EBVD, 1 R-CHOP (rituximab, cyclophosphamide, doxorubicin, vincristine, prednisone) and ABVD (Adriamycin, bleomycin, vinblastine, dacarbazine)

Other chemotherapy regimens included 6 cycles of EBVD (epirubicin, bleomycin, vinblastine, dacarbazine) and 3 cycles of AVD (Adriamycin, vinblastine, dacarbazine), received by one patient each. One patient received 5 cycles of ChIVPP (chlorambucil, vinblastine, procarbazine, prednisolone), and another received one cycle of EBVD and then three cycles of ChIVPP. Finally, one patient received three cycles of R-CHOP (rituximab, cyclophosphamide, hydroxydaunorubicin, Oncovin and prednisone) followed by three cycles of ABVD on histology review. Reasons for the use of ChlVPP were severely reduced left ventricular ejection fraction and inability to tolerate anthracycline chemotherapy. The supply of doxorubicin was limited for a short period due to logistical supply issues and Epirubicin was substituted. Bleomycin was omitted in a patient with pre-existing lung function abnormalities.

All patients with HIV were managed using the same treatment protocols as the HIV negative group. Thirteen PLWH were on ART at cHL diagnosis and the remaining six were initiated on ART prior to receiving chemotherapy. The median CD4 count at diagnosis of cHL was 311 cells/mm^3^ (IQR: 128–426 cells/mm^3^).

*Imaging to assess response to treatment*: Almost all patients were imaged using PET/CT at interim/end of treatment (93%). Three patients had CT restaging, and one patient did not need another scan because the staging PET/CT did not demonstrate evidence of active lymphoma after excision biopsy of the involved lymph node. Four patients did not have an interim or end of treatment scan. Two of these patients were fully treated but were lost to follow-up or missed their CT appointments. The other two deviated from their treatment plans as follows: a 20-year-old HIV negative male (stage II unfavourable) and a 28-year-old female with HIV (stage I unfavourable) were planned for four cycles of ABVD and ISRT, but both only received two cycles of ABVD before being lost to follow-up. These four patients were classified as having an unconfirmed complete response (CR) to treatment and were recorded to be alive and in good health at five and 12.5, and eight and 10.5 years after diagnosis, respectively.

*End of treatment response assessment:* A CR to primary treatment was documented in 86% of patients, two patients (3%) had a partial response (PR), and four patients (6%) had progressive disease (PD) (see Table 2). Three patients (4%) relapsed after primary treatment, all of whom were male, HIV negative and had NSHL. The five patients who had progressive or refractory disease were all HIV negative with stage II NSHL. Similar proportions of patients classified as NCCN favourable and unfavourable were observed across the three response groups (see Table 3).

**Table 3:** Demographic and clinical characteristics by disease status

	*N* (%) or Median (IQR)		
Variable	Patients without disease progression (*N* = 62)	Patients with progressive or refractory disease (*N* = 5)	Patients who relapsed (*N* = 3)
Sex			
Male	29 (47)	3 (60)	3 (100)
Female	33 (53)	2 (40)	0
Age (years)	36 (26–46)	32 (26–33)	35 (21–47)
Age >50 years	10 (14)	0	0
Clinical stage			
I	9 (15)	0	1 (33)
II	53 (86)	5 (100)	2 (67)
Histological classification			
NSHL	33 (53)	5 (100)	3 (100)
MCHL	15 (24)	0	0
LRHL	1 (2)	0	0
LDHL	2 (3)	0	0
HL unspecified	11 (18)	0	0
HIV status			
Negative	43 (70)	5 (100)	3 (100)
Positive	19 (31)	0	0
NCCN risk factors			
MMR >0.33	5 (8)	2 (40)	0
Bulky disease	8 (13)	2 (40)	0
ESR ≥ 50 mm/hr	18 (29)	0	1 (33)
B-symptoms present	22 (36)	3 (60)	2 (67)
>3 nodal regions involved	24 (39)	3 (60)	1 (33)
NCCN classification			
Favourable	15 (24)	1 (20)	1 (33)
Unfavourable	47 (76)	4 (80)	2 (67)

*HL* Hodgkin lymphoma, *NSHL* nodular sclerosing HL, *MCHL* mixed cellularity HL, *LRHL* lymphocyte rich HL, *LDHL* lymphocyte depleted HL, *NCCN* National Comprehensive Cancer Network, *IQR* Interquartile range, *MMR* mediastinal mass ratio, *ESR* erythrocyte sedimentation rate

### Outcome and additional associations with overall survival

**Fig. 2:**
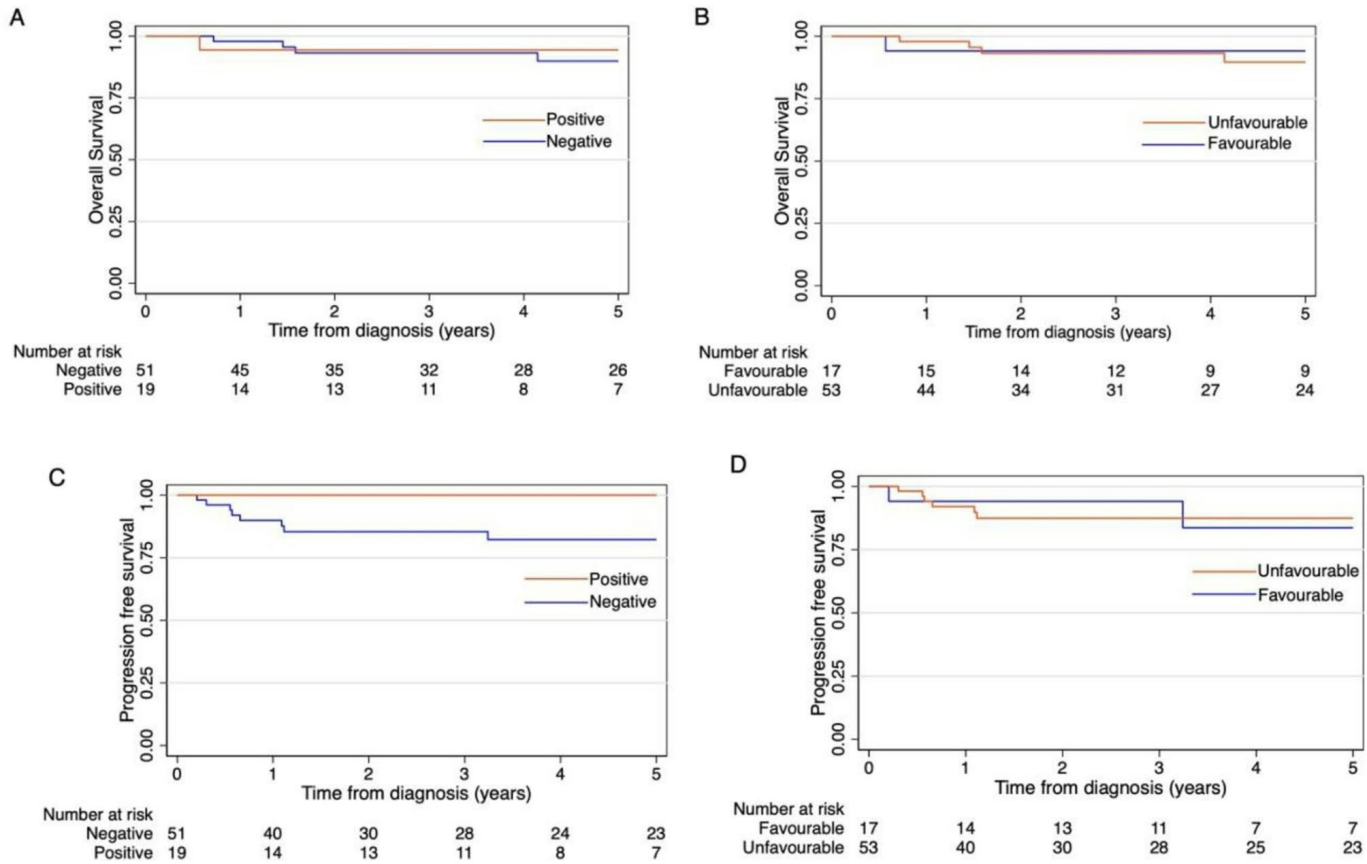
Kaplan–Meier Curves of five-year overall survival by (A) HIV status (*P* = 0.684) and (B) NCCN classification (*P* = 0.599). Progression-free survival by (C) HIV status (*P* = 0.086) and (D) NCCN classification (*P* = 0.984)

The data was evaluated for five-year OS according to HIV status and NCCN classification (Fig. 2A and B). Patients had a median follow-up duration of 4.1 years (IQR: 1.7–6.6 years). The five-year survival for the total cohort was 91% (95% CI: 78.8–96.2). The five-year survival for the HIV positive and negative groups was 94% (95% CI: 66.6–99.2) and 90% (95% CI: 74.9–96.2, *p* = 0.684), respectively. The five-year survival for the favourable group was 94% (95% CI: 65.0–99.2.0.2) and that of the unfavourable group was 90% (95% CI: 74.1–96.1, *p* = 0.599). PFS curves are shown in Fig. 2C and D. PFS at five years for the total cohort was 87% (95% CI: 74.9–93.2), while PFS at five years for the HIV positive and negative groups was 100% and 82% (95% CI: 67.2–90.8, *p* = 0.086), respectively. PFS at five years for the favourable and unfavourable groups was 84% (95% CI: 46.5–95.9), and 87% respectively (95% CI: 74.0–94.2, *p* = 0.984). At the end of the study 91% of the patients were alive, and six patients (9%) had died. Of note, only two patients (3%) died from PD, all others demised due to unrelated causes (see Table 2). In univariable and multivariate logistic regression no factors were associated with HL failure (Supplementary Table 1).

## Discussion

Patients treated in the SA public health system with early-stage cHL showed excellent patient outcomes with a 91% five-year OS, favourably aligning with outcomes from single and multicentre studies as well as national cancer registries around the world [11, 28, 29, 33, 34]. Neither HIV status nor NCCN risk stratification affected outcome. It was an unexpected observation that PLWH exhibited outcomes at least as favourable as those without HIV, with no instances of primary refractory and relapsed disease.

Five-year OS was comparable in the NCCN favourable and unfavourable risk groups (94% and 90%, respectively). Other studies from the sub-Saharan African region, could not apply precise risk stratification in early-stage cHL cohorts [11, 19, 23, 34]. This fact, as well as small early-stage cHL numbers, has precluded meaningful outcomes comparison in favourable and unfavourable cohorts being made in low-income or HIV-endemic settings. Precise staging was possible in our cohort as the majority of patients had PET/CT at diagnosis as well as for treatment assessment. Nevertheless, our study did not have sufficient patient numbers to validate the use of NCCN early-stage parameters [8, 9, 33]. While novel adverse prognostic factors may improve stratification of early stage disease in low-income settings, these are not yet available [19, 23]. The focus should remain on promoting early diagnosis and addressing the factors that lead to late diagnosis [18].

In the South African setting, patients with HIV-associated HL are more likely to present with advanced disease [14, 17]. Previous studies in patients with cHL at any disease stage in our centre showed HIV positivity conferred a worse prognosis with up to 48% of patients dying within one year of diagnosis. This despite using the same treatment regimens as high-income settings where the 5-year OS was 81% [15, 19, 35]. Another South African study and a study in Tanzania also reported worse OS in PLWH [17, 24]. In patients without bone marrow infiltration, however, PLWH were shown to have better outcomes than those without HIV [19]. This suggests advanced disease stage as a principal reason for not obtaining outcomes seen in high-income countries, where PLWH on ART have response and survival rates resembling those of HIV negative patients [35–37]. Further support for this comes from Botswana and Malawi where higher percentages of early-stage cHL and improved outcomes are seen [23, 38]. Our study demonstrated a single, non–disease-related, death in the HIV-positive early cHL cohort, with no cases of refractory or relapsed disease. These findings strongly underscore the importance of early diagnosis.

Diagnostic delay drives advanced diagnosis [15]. The initiation of empiric tuberculosis therapy due to the diagnostic challenges in differentiating lymphoma from tuberculosis, HIV and other opportunistic infections - which obscure the underlying pathology - and the lack of access to peripheral lymph node biopsy are key contributors to this delay [15, 39]. This risk was evident in our study with 40% of our cohort presenting with B symptoms and nodal involvement. These characteristics are frequently misattributed to HIV and tuberculosis in young people, in whom malignancy is often not considered as a differential diagnosis [15]. We show that in this early-stage cohort, 13% of patients were on anti-tuberculosis therapy at diagnosis, but only 5% had proven tuberculosis. This in contrast to earlier studies from our institution where up to 33% of advanced-stage patients were empirically treated for tuberculosis and 10% had proven tuberculosis [19].

This study has several limitations, mainly due to retrospective study design. This restricted both the scope and availability of patient and laboratory data (including ESR, crucial for prognostic assessment and inconsistency in the diagnostic modalities used to assess for tuberculosis). Secondly, the study’s small sample size precluded multivariate analyses. Nevertheless, the sample size should be considered in the context of the much larger total cHL cohort treated concurrently, where our ongoing research is focused on the determinants of diagnostic delay associated with poor outcomes. Lastly, the retrospective acquisition of CT and PET/CT reports introduced a potential source of variability, and despite thorough review of imaging reports, the assurance of standardized reporting could not be guaranteed uniformly across the dataset.

## Conclusion

This study focuses on the risk determinants and treatment outcomes of early-stage cHL patients, highlighting pertinent facts not yet described in the African context. We convincingly present excellent outcomes in this cohort despite the limitations experienced in our resource constrained setting. We also present excellent outcomes in PLWH treated for cHL. And we clearly present the arguement that healthcare innovation in the African setting should be focused on identifying and diagnosing patients earlier. Decreasing diagnostic delays will alter the burden of disease to align more closely with the more equal distribution of early versus advanced cHL that is seen in high income countries. We are addressing this gap in healthcare in our region with a cost-effective pathway to improve early diagnosis by reducing diagnostic delay [18]. This will ultimately result in more patients experiencing the excellent outcomes of early-stage Hodgkin disease.

## Supplementary Information

Supplementary Material 1. Supplementary Information Materials and methods: recorded data Patient sex, age, and HIV status at cHL diagnosis were documented.The date of cHL diagnosis corresponded to date of tissue diagnosis. Lymph node specimens were histologically subtyped into nodular sclerosing (NSHL), mixed cellularity (MCHL), lymphocyte rich (LRHL), lymphocyte depleted (LDHL), and HL unspecified, according to the revised 2016 World Health Organization (WHO) Classification [40]. Laboratory results included haemoglobin, albumin, white cell count, lymphocyte count and ESR at, or within, 2 weeks of diagnosis.Modified Lugano stage at diagnosis was determined by 2-deoxy-2-[^18^F]fluoro-D-glucose (2-[^18^F]FDG) positron emission tomography / computed tomography (PET/CT). CT was used if PET/CT was not performed due to logistical or resource constraints. Only patients with stage I and II cHL were included. Prognosis was determined using the NCCN classification. Early-stage disease was classified as unfavourable when any one of the following four risk factors were positive: ESR >50 or any B-symptoms; Nodal sites >3; MMR >0.33 calculated as maximum width of the mass over maximum intrathoracic diameter on chest x-ray; Any single node or nodal mass >10cm in diameter [7]. Early-stage favourable disease exhibited no risk factors.Investigations pertaining to the co-diagnosis of tuberculosis that were analysed included: testing with smears for acid-fast bacilli, GeneXpert, tuberculosis culture and assessment for caseous granulomata on various sample types (sputum, blood, nodal or extra-nodal tissue, bone marrow aspirate and trephine, and fine-needle aspirates).

## Data Availability

The dataset used and analysed during the current study are available from the corresponding author on reasonable request.

## Abbreviations

2-[^18^F]FDG: 2-deoxy-2-[^18^F]fluoro-D-glucose
ABVD: Adriamycin, bleomycin, vinblastine, dacarbazine
ART: Antiretroviral therapy
AVD: Adriamycin, vinblastine, dacarbazine
BEACOPP: Bleomycin, etoposide, Adriamycin, cyclophosphamide, Oncovin, procarbazine, prednisone
ChIVPP: Chlorambucil, vinblastine, procarbazine, prednisolone
cHL: Classical HL
CR: Complete response
DS: Deauville score
EBVD: Epirubicin, bleomycin, vinblastine, dacarbazine
EORTC: European Organization for Research and Treatment of Cancer
ESMO: European Society for Medical Oncology
ESR: Erythrocyte sedimentation rate
GHSG: German Hodgkin Study Group
GSH: Groote Schuur Hospital
HIV: Human immunodeficiency virus
HL: Hodgkin Lymphoma
HPR: Haematology Patient Registry
ILROG: International Lymphoma Radiation Oncology Group
IQR: Interquartile range
ISRT: Involved site radiation therapy
LDHL: Lymphocyte depleted Hodgkin lymphoma
LRHL: Lymphocyte rich Hodgkin lymphoma
MCHL: Mixed cellularity Hodgkin lymphoma
MMR: Mediastinal mass ratio
NCCN: National Comprehensive Cancer Network
NHL: Non-Hodgkin lymphoma
NHLS: National Health Laboratory Service
NSHL: Nodular sclerosing Hodgkin lymphoma
OS: Overall survival
PD: Progressive disease
PET/CT: Positron emission tomography / computed tomography
PFS: Progression-free survival
PLWH: People living with HIV
PR: Partial response
R-CHOP: Rituximab, cyclophosphamide, hydroxydaunorubicin, Oncovin and prednisone
SA: South Africa
WHO: World Health Organization
