# Correction to: Early-stage hodgkin lymphoma in Cape Town, South Africa: prognostic risk factors at diagnosis and treatment outcomes

**DOI:** 10.1186/s12885-025-15452-7

**Published:** 2026-01-21

**Authors:** Shakira Dawood, Brigid McMillan, Karryn Brown, Jenna Oosthuizen, David Richardson, Kudakwashe Simba, Lillian F. Andera, Jessica Opie, Katherine Antel, Stuart More, Ayesha Allie, Zainab Mohamed, Estelle Verburgh

**Affiliations:** 1 Department of Medicine, University of Cape Town and Groote Schuur Hospital, Cape Town, South Africa; 2 Department of Radiation Oncology, University of Cape Town and Groote Schuur Hospital, Cape Town, South Africa; 3 Department of Medicine, Division of Clinical Haematology, University of Cape Town and Groote Schuur Hospital, Cape Town, South Africa; 4 Department of Pathology, Division of Haematology, National Health Laboratory Service, University of Cape Town and Groote Schuur Hospital, Cape Town, South Africa; 5 Division of Haematology and Oncology, Medical University of South Carolina, Charleston, USA; 6 Department of Radiation Medicine, Division of Nuclear Medicine, University of Cape Town and Groote Schuur Hospital, Cape Town, South Africa

**Correction to: BMC Cancer 25, 1813 (2025)**

**https://doi.org/10.1186/s12885-025-15219-0**

Following publication of the original article [1], the authors reported an error in Abstract section, Keywords, Background section, Results section, Discussion section, Table 1, and Table 3.

Abstract section.

- Background
  - “…uncommon in these setting” should be change to “…uncommon in these settings”.

- Results
  - (P = 0.86) should be change to (P = 0.086).

- Conclusions
  - “…disease stages, highlights the” should be change to “…disease stages, we highlight the”.

Keywords

“South africa” should be change to “South Africa”.

Background section

- Last sentence of the 2nd paragraph.

“…we reported 5-year **OS** of 56% and” should be change to “we reported 5-year **define OS** of 56% and”.

- Last paragraph.

"The aim of this retrospective study **is** to focus on the early-stage patient subset of the consecutive cHL cohort diagnosed and treated in our centre since 2010. We sought to determine progression-free survival (PFS) and OS in this subset and how this **correlates** with the NCCN staging system and its individual risk factors. We **hypothesise** that if early-stage cHL patient outcomes in our setting prove to be internationally comparable, it confirms that our primary priority for obtaining cure in cHL must be to address healthcare system delays” should be change to “The aim of this retrospective study **was** to focus on the early-stage patient subset of the consecutive cHL cohort diagnosed and treated in our centre since 2010. We sought to determine progression-free survival (PFS) and OS in this subset and how this **correlated** with the NCCN staging system and its individual risk factors. We **hypothesised** that if early-stage cHL patient outcomes in our setting prove to be internationally comparable, it confirms that our primary priority for obtaining cure in cHL must be to address healthcare system delays."

Results section.

- Study population section (2nd paragraph, 2nd sentence).

“all of whom were (**7%** of the” should be change to “all of whom were (**7% PLWH** of the”.

- Treatment section (last paragraph).

“End of treatment response assessment” needs to be in italics with a semi colon after assessment.

- Outcome and additional associations with overall survival section.
  - “p = 0.86” should be change to “p = 0.086”;
  - “CI: 74.0–**94.2.0.2**,” should be change to “CI: 74.0–**94.2**,”

Discussion section.

- 3rd paragraph.

…high-income **setting** where” should be change to “…high-income **settings** where.

- 4th paragraph, last sentence.

…tuberculosis **therapy** and 10%” should be change to “tuberculosis and 10%.

Table 1.

- The highlighted “.**9**” in White cell count (x109/L) row should be change to “**9**”.

Incorrect


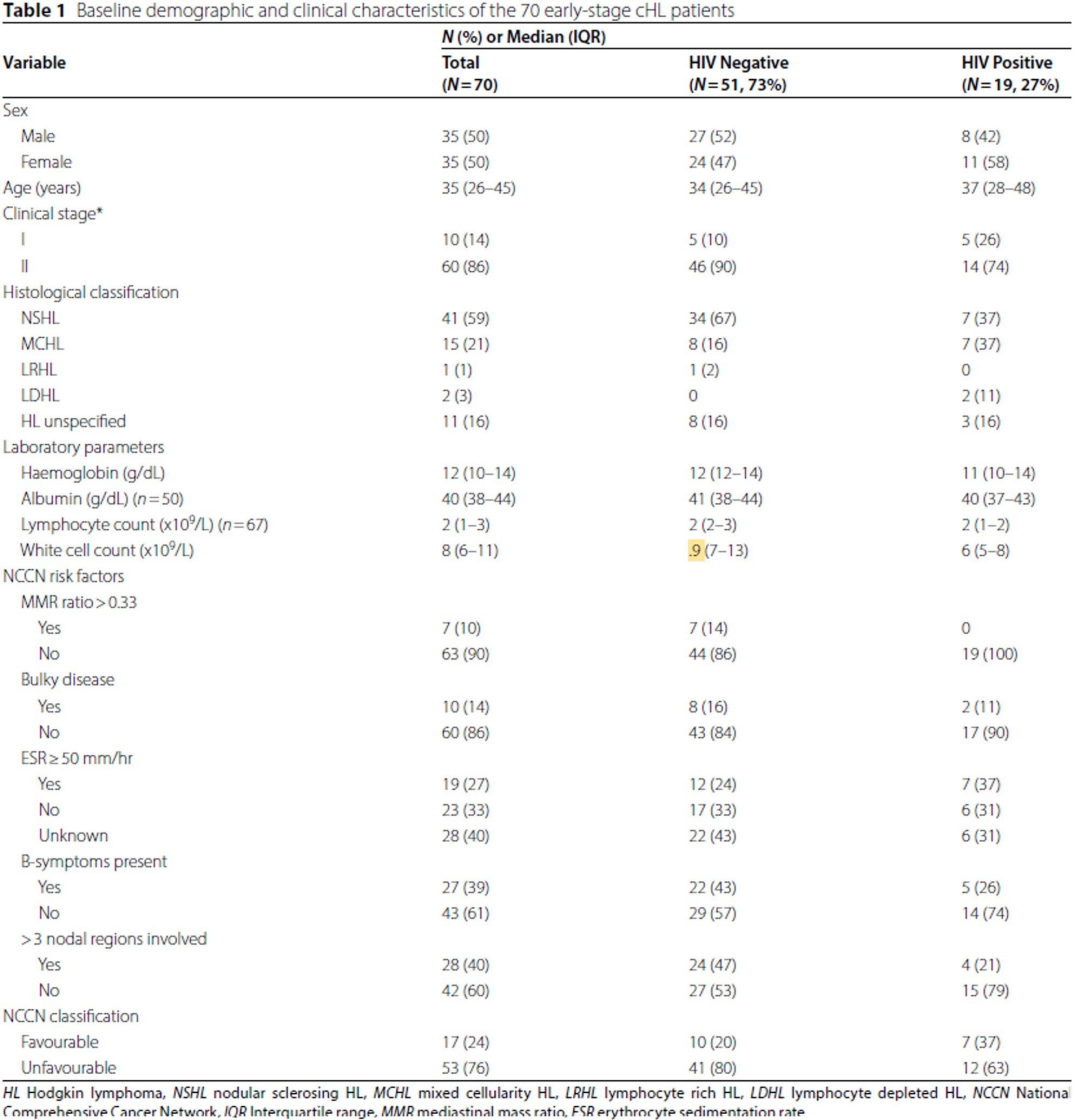


Corrected

**Table 1:** Baseline demographic and clinical characteristics of the 70 early-stage cHL patients

	**N (%) or Median (IQR)**		
**Variable**	**Total** (N=70)	**HIV Negative** (N=51, 73%)	**HIV Positive** (N=19, 27%)
**Sex**			
Male	35 (50)	27 (52)	8 (42)
Female	35 (50)	24 (47)	11 (58)
**Age **(years)	35 (26-45)	34 (26-45)	37 (28-48)
Clinical stage*			
I	10 (14)	5 (10)	5 (26)
II	60 (86)	46 (90)	14 (74)
**Histological classification**			
NSHL	41 (59)	34 (67)	7 (37)
MCHL	15 (21)	8 (16)	7 (37)
LRHL	1 (1)	1 (2)	0
LDHL	2 (3)	0	2 (11)
HL unspecified	11 (16)	8 (16)	3 (16)
**Laboratory parameters **			
Haemoglobin (g/dL)	12 (10-14)	12 (12-14)	11 (10-14)
Albumin (g/dL) (n=50)	40 (38-44)	41 (38-44)	40 (37-43)
Lymphocyte count (x10^9^/L) (n=67)	2 (1-3)	2 (2-3)	2 (1-2)
White cell count (x10^9^/L)	8 (6-11)	9 (7-13)	6 (5-8)
**NCCN risk factors**			
**MMR ratio >0.33**			
Yes	7 (10)	7 (14)	0
No	63 (90)	44 (86)	19 (100)
**Bulky disease**			
Yes	10 (14)	8 (16)	2 (11)
No	60 (86)	43 (84)	17 (90)
**ESR ≥50 mm/hr**			
Yes	19 (27)	12 (24)	7 (37)
No	23 (33)	17 (33)	6 (31)
Unknown	28 (40)	22 (43)	6 (31)
**B-symptoms present**			
Yes	27 (39)	22 (43)	5 (26)
No	43 (61)	29 (57)	14 (74)
**>3 nodal regions involved**			
Yes	28 (40)	24 (47)	4 (21)
No	42 (60)	27 (53)	15 (79)
**NCCN classification **			
Favourable	17 (24)	10 (20)	7 (37)
Unfavourable	53 (76)	41 (80)	12 (63)

*HL *Hodgkin lymphoma, *NSHL *Nodular sclerosing, *HL *MCHL Mixed cellularity, *HL *LRHL Lymphocyte rich HL, *LDHL *Lymphocyte depleted HL, *NCCN *National

Table 3.

- Remove extra blank entry between Age (years) and Age > 50 years;
- The last column width needs to be fixed, numbers are spilling over into incorrect rows;
- 35 (21–47) should be in the same line.

Incorrect


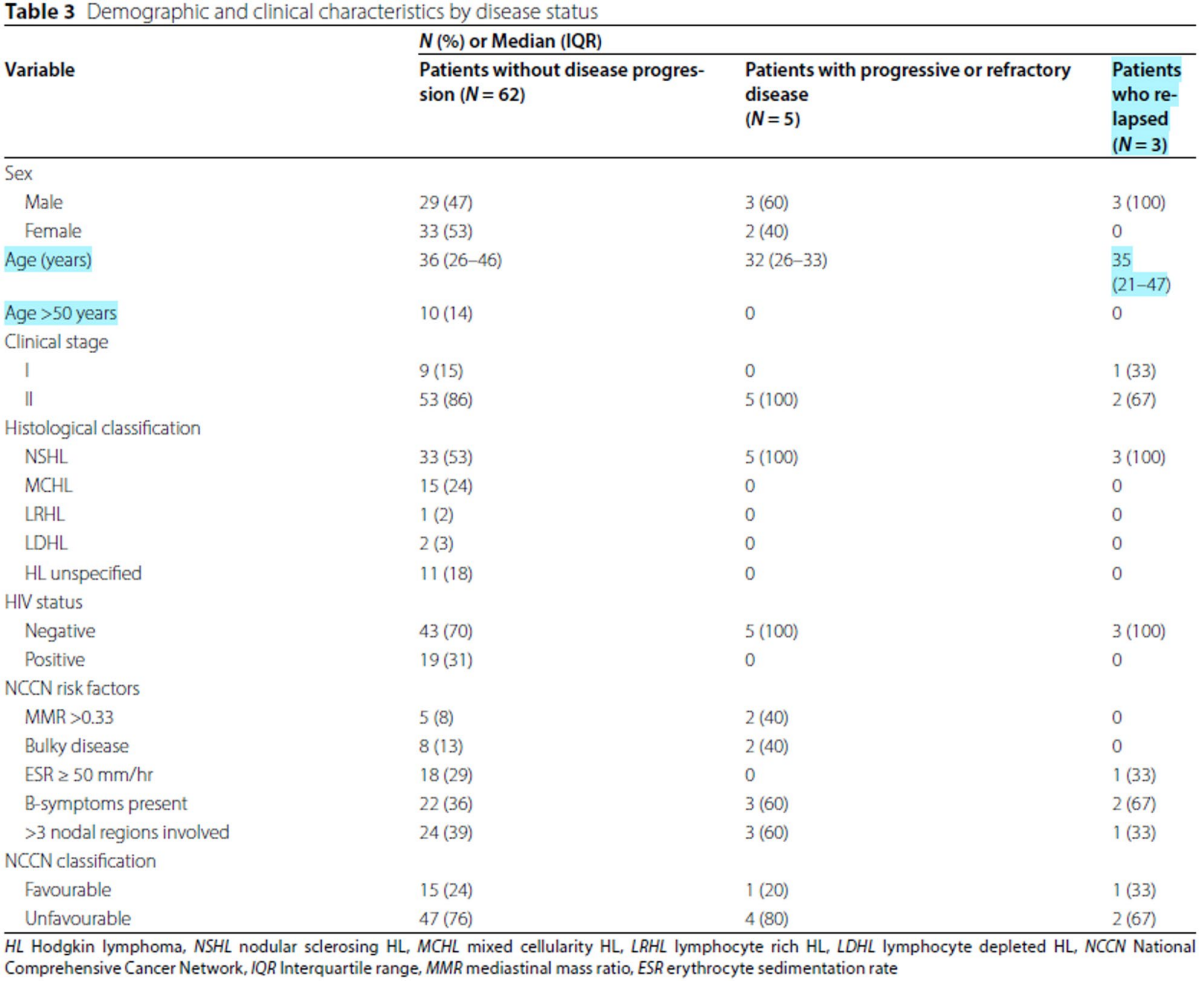


Correct

**Table 3:** Demographic and clinical characteristics by disease status

	**N (%), Median (IQR) or Mean (SD)**		
**Variable**	**Patients without disease progression (N=62)**	**Patients with progressive or refractory disease** **(N=5)**	**Patients who relapsed (N=3)**
**Sex**			
Male	29 (47)	3 (60)	3 (100)
Female	33 (53)	2 (40)	0
**Age **(years)	36 (26-46)	32 (26-33)	35 (21-47)
**Age > 50 years**	10 (14)	0	0
**Clinical stage**			
I	9 (15)	0	1 (33)
II	53 (86)	5 (100)	2 (67)
**Histological classification**			
NSHL	33 (53)	5 (100)	3 (100)
MCHL	15 (24)	0	0
LRHL	1 (2)	0	0
LDHL	2 (3)	0	0
HL unspecified	11 (18)	0	0
**HIV status **			
Negative	43 (70)	5 (100)	3 (100)
Positive	19 (31)	0	0
**NCCN risk factors**			
MMR >0.33	5 (8)	2 (40)	0
Bulky disease	8 (13)	2 (40)	0
ESR ≥50 mm/hr	18 (29)	0	1 (33)
B-symptoms present	22 (36)	3 (60)	2 (67)
>3 nodal regions involved	24 (39)	3 (60)	1 (33)
**NCCN classification **			
Favourable	15 (24)	1 (20)	1 (33)
Unfavourable	47 (76)	4 (80)	2 (67)
