# Supplementary material for: Early-stage Hodgkin lymphoma in Cape Town, South Africa: prognostic risk factors at diagnosis and treatment outcomes
Source: BMC Cancer. 2025 Nov 25;25:1813. doi: 10.1186/s12885-025-15219-0 (PMC12645670; doi:10.1186/s12885-025-15219-0)
Supplement: Supplementary file 1 — Supplementary Material 1. Supplementary Information Materials and methods: recorded data Patient sex, age, and HIV status at cHL diagnosis were documented.The date of cHL diagnosis corresponded to date of tissue diagnosis. Lymph node specimens were histologically subtyped into nodular sclerosing (NSHL), mixed cellularity (MCHL), lymphocyte rich (LRHL), lymphocyte depleted (LDHL), and HL unspecified, according to the revised 2016 World Health Organization (WHO) Classification [40]. Laboratory results included haemoglobin, albumin, white cell count, lymphocyte count and ESR at, or within, 2 weeks of diagnosis.Modified Lugano stage at diagnosis was determined by 2-deoxy-2-[18F]fluoro-D-glucose (2-[18F]FDG) positron emission tomography / computed tomography (PET/CT). CT was used if PET/CT was not performed due to logistical or resource constraints. Only patients with stage I and II cHL were included. Prognosis was determined using the NCCN classification. Early-stage disease was classified as unfavourable when any one of the following four risk factors were positive: ESR >50 or any B-symptoms; Nodal sites >3; MMR >0.33 calculated as maximum width of the mass over maximum intrathoracic diameter on chest x-ray; Any single node or nodal mass >10cm in diameter [7]. Early-stage favourable disease exhibited no risk factors.Investigations pertaining to the co-diagnosis of tuberculosis that were analysed included: testing with smears for acid-fast bacilli, GeneXpert, tuberculosis culture and assessment for caseous granulomata on various sample types (sputum, blood, nodal or extra-nodal tissue, bone marrow aspirate and trephine, and fine-needle aspirates). [file 12885_2025_15219_MOESM1_ESM.docx]

Supplementary Table 1. Patient Treatment According to Disease Stage (7, 27)

| Stage of Disease | Treatment |
| --- | --- |
| Early-stage | Combined (chemotherapy and ISRT)  OR  Chemotherapy only |
| Very good risk | 2 cycles ABVD  AND  20Gy ISRT |
| Intermediate / poor risk early-stage | 4 cycles ABVD  AND  30Gy ISRT |

Supplementary Table 2. Analyses of factors associated with HL failure (disease progression/refractory disease/relapse)

|  | **Univariate** | | | **Multivariate** | | |
| --- | --- | --- | --- | --- | --- | --- |
| **Variable** | **Odds ratio** | **95% CI** | **P-value** | **Odds ratio** | **95% CI** | **P-value** |
| Age | 0.96 | 0.89-1.02 | 0.204 | 0.95 | 0.88-1.03 | 0.256 |
| Clinical stage II | 1.19 | 0.13-10.85 | 0.878 | 0.64 | 0.05-7.64 | 0.726 |
| NCCN unfavourable | 0.96 | 0.17-5.25 | 0.960 | * |  |  |
| MMR >0.33 | 3.80 | 0.60-24.00 | 0.156 | 1.69 | 0.13-22.0 | 0.689 |
| Bulky disease | 2.25 | 0.39-13.13 | 0.368 | 1.35 | 0.12-15.39 | 0.808 |
| ESR | 1.00 | 0.97-1.04 | 0.834 | ** |  |  |
| ESR ≥50 mm/hr | 1.22 | 0.07-20.94 | 0.890 | ** |  |  |
| B symptoms | 3.03 | 0.66-13.90 | 0.154 | 2.76 | 0.56-13.70 | 0.214 |
| > 3 nodal regions | 1.58 | 0.36-6.94 | 0.542 | 1.31 | 0.27-6.37 | 0.737 |

* NCCN classification omitted due to collinearity with factors included in the classification system, which were included in the model (MMR, bulky disease, B symptoms, nodal regions)

** ESR excluded from model as it was only available for 42 patients


All patients who progressed were younger than 50 and were HIV negative, therefore these variables could not be evaluated in logistic regression

**Supplementary Information**

**Materials and methods: recorded data**

Patient sex, age, and HIV status at cHL diagnosis were documented. The date of cHL diagnosis corresponded to date of tissue diagnosis. Lymph node specimens were histologically subtyped into nodular sclerosing (NSHL), mixed cellularity (MCHL), lymphocyte rich (LRHL), lymphocyte depleted (LDHL), and HL unspecified, according to the revised 2016 World Health Organization (WHO) Classification (40). Laboratory results included haemoglobin, albumin, white cell count, lymphocyte count and ESR at, or within, 2 weeks of diagnosis.

Modified Lugano stage at diagnosis was determined by 2-deoxy-2-[^18^F]fluoro-D-glucose (2-[^18^F]FDG) positron emission tomography / computed tomography (PET/CT). CT was used if PET/CT was not performed due to logistical or resource constraints. Only patients with stage I and II cHL were included.

Prognosis was determined using the NCCN classification. Early-stage disease was classified as unfavourable when any one of the following four risk factors were positive: ESR >50 or any B-symptoms; Nodal sites >3; MMR >0.33 calculated as maximum width of the mass over maximum intrathoracic diameter on chest x-ray; Any single node or nodal mass >10cm in diameter (7). Early-stage favourable disease exhibited no risk factors.

Investigations pertaining to the co-diagnosis of tuberculosis that were analysed included: testing with smears for acid-fast bacilli, GeneXpert, tuberculosis culture and assessment for caseous granulomata on various sample types (sputum, blood, nodal or extra-nodal tissue, bone marrow aspirate and trephine, and fine-needle aspirates).
